# Closing the gap on injury prevention: the Oslo Sports Trauma Research Centre four-platform model for translating research into practice

**DOI:** 10.1136/bjsports-2021-104998

**Published:** 2022-02-07

**Authors:** Hege Heiestad, Grethe Myklebust, Kaja Funnemark, Christine Holm Moseid

**Affiliations:** Department of Sports Medicine, Norwegian School of Sports Sciences, Oslo, Norway

**Keywords:** sporting injuries, sports, sports medicine

Multiple studies across multiple sports have demonstrated that tailored exercise programmes reduce the risk of sport injuries by about 50%.^1–3^ These programmes typically include exercises to improve balance and neuromuscular control, optimise landing and turning techniques, and increase muscle strength and endurance.

## The ‘proof is in the pudding’

Despite the well-documented positive effects on injury risk,^1–3^ far too few athletes implement injury prevention exercises and programmes in their daily training.^4–6^ Real-life implementation in the field is poor, with high injury rates across most sports as a consequence. Medical personnel around the world are concerned because sports injuries represent a major reason why many children and youth quit sport. They may develop a more sedentary lifestyle, with higher health burdens and costs to both society and the individual.^7^ Today, organised sports represent the most important source for promoting good health through physical activity in children and youth.^8^ Reducing the risk of injury from sports participation should, therefore, be a priority for all stakeholders including medical professionals, coaches, strength and conditioning specialists, parents and athletes.

In order to obtain the cost-effective effects from injury prevention training, it is important to successfully implement these programmes at the grass roots level, reaching the majority of young individuals active in sports. There is an urgent need to translate science into action and increase everyday injury prevention training for children and youth across all sports, by reaching out and involving coaches, athletes and parents, as well as key stakeholders in sport at the organisational level. The goal is to establish good training routines from an early age, with preventive programmes as a natural, integrated part of practice and training.

## The Oslo Sports Trauma Research Centre four-platform model for translating research into practice

The Oslo Sports Trauma Research Centre was established in May 2000 as an interdisciplinary research centre focusing on methods to prevent sports injuries (and other health problems) with both sports and medical expertise and as the hub in the wheel for a national research network. In collaboration with the International Olympic Committee, we developed the free mobile application Skadefri (Get Set) and the website www.skadefri.no (fittoplay.org) to translate science into action, spreading the word on the significant potential benefits from injury prevention training at the individual, team, club and societal level.

**Figure 1:**
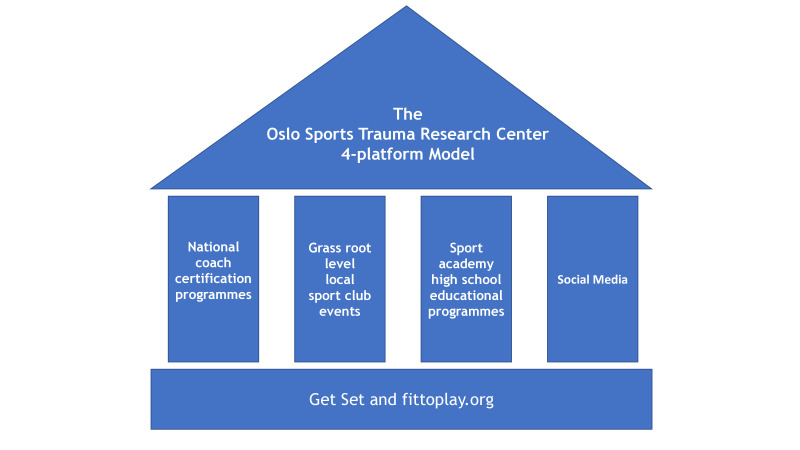
The Oslo Sports Trauma Research Centre four-platform model for translating research into practice.

During the past 2 years, we have identified four main focus areas to promote knowledge translation, as described below (figure 1).

### National coach certification programmes

Coaches play a key role in the implementation of injury prevention. We have developed a 2-hour e-learning course, ‘Sports without injuries’, discussing the most important aspects of injury prevention and injury prevention training. This is a basic course, directed at all coaches, particularly for children and youth. We will also develop further sport-specific educational in-person workshops for coaches, to increase their knowledge and confidence in planning and conducting injury prevention training for their athletes.

### Sport club events

These 2-hour in-person workshops aim to reach coaches, athletes, parents and sports club staff at the local, grass roots level. They are held by Skadefri-educated sports physiotherapists and physicians throughout the country, delivered according to a standardised template, covering both the theoretical background as well as practical injury prevention sessions for everyday training. The local sport club events contribute towards creating a national sport movement that is safer, more sustainable and inclusive by creating safer sports organisations and environments for athletes involved at all levels.

### Sport academy high school educational programmes

Our national Olympic committee recognises specialised national sport academy high schools to develop talented youth elite athletes at the highest level. Through a 10-hour specialised educational programme, our aim is to create more resilient youth elite athletes. This programme is athlete-centred and includes practical and theoretical sessions on relevant sport-specific physiological and psychological demands with modules adapted to each of the grade levels from 8th through 13th grade. The programme is anchored within the national educational school system with a commitment from management and coaches at each school.

### Social media (some)

The SoMe channels give us the opportunity to translate and produce credible, engaging and relevant content of value for the sporting community at all levels. Instagram and Facebook are our main channels. Many small inputs over time, on different areas, increases knowledge and the likelihood of behavioural change through adoption and maintenance of injury prevention training. Through SoMe, we reach our target groups directly in a natural, unforced and self-determined setting. We use videos, exercises and ‘did you know’—posts to educate and enhance knowledge on injury prevention by translating research into practice in an informal and entertaining way.

Skadefri (Get Set) aims to contribute to promote sport for all and to the fundamental principles in the field of sport and education, by supporting athlete health and integrity at all levels of sport. It promotes safe sport, educates young people, their coaches and their parents and addresses issues of safeguarding, healthy lifestyles and injury prevention training. We believe the combination of e-learning, in-person grass roots workshops, specialised programmes where appropriate, and engaging SoMe content provide the framework to increase implementation and close the gap on injury prevention.
